# Quality of Dietary Fat Intake and Body Weight and Obesity in a Mediterranean Population: Secondary Analyses within the PREDIMED Trial

**DOI:** 10.3390/nu10122011

**Published:** 2018-12-19

**Authors:** Yvette Beulen, Miguel A. Martínez-González, Ondine van de Rest, Jordi Salas-Salvadó, José V. Sorlí, Enrique Gómez-Gracia, Miquel Fiol, Ramón Estruch, José M. Santos-Lozano, Helmut Schröder, Angel Alonso-Gómez, Luis Serra-Majem, Xavier Pintó, Emilio Ros, Nerea Becerra-Tomas, José I. González, Montserrat Fitó, J. Alfredo. Martínez, Alfredo Gea

**Affiliations:** 1 Division of Human Nutrition, Wageningen University, 6708 Wageningen, The Netherlands; yvette.beulen@wur.nl (Y.B.); ondine.vanderest@wur.nl (O.v.d.R.); 2 Department of Preventive Medicine & Public Health, University of Navarra, 31008 Pamplona, Spain; mamartinez@unav.es; 3 CIBER Fisiopatología de la Obesidad y Nutrición (CIBEROBN), Instituto de Salud Carlos III (ISCIII), Spanish Government, 28029 Madrid, Spain; jordi.salas@urv.cat (J.S.-S.); sorli@uv.es (J.V.S.); mfiol@hsd.es (M.F.); restruch@clinic.cat (R.E.); josemanuel.santos@ono.com (J.M.S.-L.); angelmago13@gmail.com (A.A.-G.); lluis.serra@ulpgc.es (L.S.-M.); xpinto@bellvitgehospital.cat (X.P.); eros@clinic.cat (E.R.); nerea.becerra@urv.cat (N.B.-T.); arraez@uv.es (J.I.G.); mfito@imim.es (M.F.); jalfmtz@unav.es (J.A.M.); 4 Human Nutrition Unit, Faculty of Medicine and Health Sciences, Pere Virgili Health Research Institute, Rovira i Virgili University, 43002 Reus, Spain; 5 Department of Preventive Medicine, University of Valencia, 46010 Valencia, Spain; 6 Department of Preventive Medicine, University of Málaga, 29016 Málaga, Spain; egomezgracia@uma.es; 7 Institute of Health Sciences IUNICS, University of Balearic Islands and Hospital Son Espases, 07010 Palma de Mallorca, Spain; 8 Department of Internal Medicine, Department of Endocrinology and Nutrition Biomedical Research Institute August Pi Sunyer (IDI- BAPS), Hospital Clinic, University of Barcelona, 08036 Barcelona, Spain; 9 Department of Family Medicine, Research Unit, Distrito Sanitario Atención Primaria Sevilla, Centro de Salud Universitario San Pablo, 41013 Sevilla, Spain; 10 Cardiovascular and Nutrition Research Group (Regicor Study Group), Hospital del Mar Research Institute (IMIM), 08003 Barcelona, Spain; hschoeder@imim.es; 11 CIBER Epidemiología y Salud Pública (CIBERESP), Instituto de Salud Carlos III (ISCIII), Spanish Government, 28029 Madrid, Spain; 12 Department of Cardiology, University Hospital of Alava, 48940 Vitoria, Spain; 13 Research Institute of Biomedical and Health Sciences (IUIBS), University of Las Palmas de Gran Canaria and Service of Preventive Medicine, Complejo Hospitalario Universitario Insular Materno Infantil (CHUIMI), Canary Health Service, 35016 Las Palmas de Gran Canaria, Spain; 14 Lipids and Vascular Risk Unit, Internal Medicine, Hospital Universitario de Bellvitge, Hospitalet de Llobregat, 08907 Barcelona, Spain; 15 Lipid Clinic, Department of Endocrinology and Nutrition Biomedical Research Institute August Pi Sunyer (IDIBAPS), Hospital Clinic, University of Barcelona, 08036 Barcelona, Spain; 16 Department of Nutrition and Food Sciences and Physiology, University of Navarra, 31008 Pamplona, Spain

**Keywords:** fat, obesity, body weight, cohort study, substitution models

## Abstract

A moderately high-fat Mediterranean diet does not promote weight gain. This study aimed to investigate the association between dietary intake of specific types of fat and obesity and body weight. A prospective cohort study was performed using data of 6942 participants in the PREDIMED trial, with yearly repeated validated food-frequency questionnaires, and anthropometric outcomes (median follow-up: 4.8 years). The effects of replacing dietary fat subtypes for one another, proteins or carbohydrates were estimated using generalized estimating equations substitution models. Replacement of 5% energy from saturated fatty acids (SFA) with monounsaturated fatty acids (MUFA) or polyunsaturated fatty acids (PUFA) resulted in weight changes of −0.38 kg (95% Confidece Iinterval (CI): −0.69, −0.07), and −0.51 kg (95% CI: −0.81, −0.20), respectively. Replacing proteins with MUFA or PUFA decreased the odds of becoming obese. Estimates for the daily substitution of one portion of red meat with white meat, oily fish or white fish showed weight changes up to −0.87 kg. Increasing the intake of unsaturated fatty acids at the expense of SFA, proteins, and carbohydrates showed beneficial effects on body weight and obesity. It may therefore be desirable to encourage high-quality fat diets like the Mediterranean diet instead of restricting total fat intake.

## 1. Introduction

Obesity is one of the most important risk factors of noncommunicable diseases. The epidemic has been described extensively in scientific literature, providing cogent evidence for its role in the development of cardiovascular diseases, diabetes, musculoskeletal disorders, and certain types of cancer [1]. Dietary fat intake has long been believed to be an important predictor of obesity as it is the most energy-dense macronutrient, providing about 9 kcal per gram versus 4 kcal/g from proteins or carbohydrates. Consequently, to control obesity, dietary guidelines have focused on reducing total fat intake for almost four decades [2]. Even after a large body of evidence showed the beneficial health effects of unsaturated fatty acids [3,4], total fat restrictions remained [5], as well as the gradual increase in the global prevalence of overweight and obesity [6].

Currently, some aspects of guidelines are being revised. A report of the third FAO (Food and Agriculture Organization of the United Nations)/WHO (World Health Organization) Expert Consultation on Fats and Fatty Acids in Human Nutrition (Geneva, 2008) showed the focus is clearly shifting from fat quantity to quality [7]. Additionally, the latest US Dietary Guidelines Advisory Committee (DGAC) report concluded that emphasis should be put on optimizing types of dietary fat and not reducing total fat [5]. The quality of fat is generally specified by the relative intake of monounsaturated (MUFA) and polyunsaturated fatty acids (PUFA) versus saturated (SFA) and trans fatty acids. Furthermore, PUFA may be subdivided into plant-derived linoleic (C18:2n-6) and α-linolenic acid (ALA, C18:3n-3) and marine-derived eicosapentaenoic (EPA, 20:5n-3) and docosahexaenoic acid (DHA, C22:6n-3) [8].

The Mediterranean diet (MeDiet) is typically high in fat and there is evidence about its role in the prevention of cardiovascular disease [9,10], in which the proportions of unsaturated and saturated fatty acids have been shown to play an important role [11]. Moreover, multiple systematic reviews and meta-analyses have shown a MeDiet does not promote weight gain [12,13,14,15]. In fact, it may even result in greater and more long-lasting weight loss than low-fat diets and be protective against increases in waist circumference [12,13,14,15]. This outcome has also been shown for nut intake, a predominant food product in the MeDiet [16,17,18]. Other food, more specific components of the MeDiet, and their association with obesity-related outcomes have not received as much attention to date.

Although assessing a complete dietary pattern provides insight into the cumulative effects of consuming certain products, determining which nutrients or food items induce the greatest change may lead to better understanding of the associations. The main objective of this study is therefore to investigate the association between dietary fat intake of both specific types of fatty acids and of food items within the MeDiet and body weight and obesity.

## 2. Materials and Methods

### 2.1. Study Population

This prospective study was a secondary analysis using data of the large, randomized controlled trial PREDIMED (prevención con dieta Mediterránea, prevention with Mediterranean diet). Participants within the PREDIMED study were Spanish men and women at high risk for cardiovascular disease, recruited from multiple centers across the country between October 2003 and June 2009. After inclusion, participants were assigned to two groups receiving a MeDiet supplemented with either extra virgin olive oil or mixed nuts, and—in the case of the control group—solely receiving advice on following a low-fat diet. The PREDIMED protocol was approved by institutional review boards and all participants provided written informed consent. Further details on the design and protocol of the study have been described elsewhere [19,20].

The trial was stopped in December 2010 and included 7447 participants. After exclusion of participants without food-frequency questionnaire (FFQ) data, without BMI follow-up data, with implausible energy intakes (<800 or >4200 kcal/day in men <500 and >3500 kcal/day in women) [21] or without other data necessary to perform the analyses, the population available for the present analyses included 6942 participants with a median follow-up of 4.8 years (interquartile range: 2.9 to 5.8).

### 2.2. Dietary Exposure Assessment

At baseline and yearly thereafter, a semiquantitative 137-item food-frequency questionnaire (FFQ) was collected and reviewed by trained, registered dieticians. The used FFQ was repeatedly validated and found to be highly reproducible and have a reasonably good validity with regard to nutrient intake reported [22,23]. Spanish food-composition tables were used to derive energy and nutrient intake [24]. Dietary intakes were considered as the cumulative average of all available data, from baseline to each follow-up measurement, to optimally represent long-term intake and reduce measurement error [25].

### 2.3. Outcome Assessment

Anthropometric measurements were taken by trained personnel once a year. Weight was measured on a calibrated scale, without shoes and with light clothing. Height was determined using a wall-mounted stadiometer.

Participants with a BMI ≥25 and <30 kg/m^2^ were classified as overweight and ≥30 kg/m^2^ as obese. Substantial weight gain or loss was defined as a change ≥10% when compared to baseline body weight [26]. Body weight was analyzed as continuous outcomes and dichotomized using this cut-off (a change ≥10%), additional to the dichotomous variables for incidence (increasing to a BMI ≥30 kg/m^2^) and reversion of obesity (decreasing to a BMI <30 kg/m^2^).

### 2.4. Assessment of Other Variables

At each yearly visit, sociodemographic variables and risk factors like educational level and working, marital, and smoking status were assessed using in a general questionnaire. Physical activity was assessed using the Spanish version of Minnesota Leisure-Time Physical Activity, validated in both sexes [27,28]. Based on frequency and duration of 67 activities, a MET (Metabolic equivalents) score was assigned to each participant. The analyses considered updated the measurements of these covariates and in case of missing data, the last observations were carried forward to prevent participants being excluded from the analyses.

### 2.5. Statistical Analyses

Participant characteristics were reported according to extreme quintiles of fatty acid intakes, as mean (SD) for continuous variables and % for categorical variables.

Generalized estimating equations (GEE) were fit to analyze the association between the cumulative average of yearly measured consumption of dietary fatty acids and body weight. For these continuous outcomes, the distribution was set to Gaussian and link function to identity. Effects of isocaloric substitution of fat types for one another, carbohydrates, and proteins were estimated as the difference between β coefficients of macronutrients exchanged per 5% of energy from these sources. Logistic GEE regression models were used to determine odds ratios for incidence and reversion of obesity and ≥10% changes in body weight. The effects of replacing specific high-fat food products for other, higher-quality options were estimated using non-isocaloric substitution models, by considering the replacement of single portions of meat, fish and seafood (100 g), oils, butter and margarine (10 g), and nuts (30 g). For all GEE models, an exchangeable correlation structure was assumed.

The model included intakes of all energy delivering dietary variables except for one (the last one being omitted as they add up to 100% for any person) and was adjusted for age, sex, intervention group, baseline BMI or body weight, leisure-time physical activity (quintiles of MET-min/week), smoking status (never, former, current smoker), educational level (none/primary, secondary, academic/graduate), working status (employed, unemployed, housewife, retired), marital status (single/widowed, married), and dietary fiber intake (continuous).

Sensitivity analyses were performed using mixed-effects models instead of GEE models and both observed and updated measurements of exposure instead of cumulative averages. Missing values of dietary variables and weight were imputed using multilevel multiple imputation in the GEE model with body weight as the continuous outcome. Data were assumed to be MAR (missing at random) and m = 20 datasets were created using multivariate normal (MVN) as the imputation algorithm [29].

All statistical analyses were performed using STATA/SE V.12.1 (StataCorp, College Station, TX, USA) and *p*-values < 0.05 were considered statistically significant.

## 3. Results

### 3.1. Baseline Characteristics

The population available for the analyses consisted of 6942 participants with a mean age of 67 (SD: 6) years and of which a proportion of 47% was obese at baseline. Table 1 shows the baseline characteristics of participants. Mean total fat intake was 30.5% of total energy intake (EN%) in the lowest quintile versus 50.6 EN% in the highest.

### 3.2. Substitution of Fat Subtypes

#### 3.2.1. Body Weight Differences

The results of GEE substitution models presented in Table 2 show the predicted effects of replacing 5% of energy from a specific fat subtype for 5 EN% of another fat subtype, proteins or carbohydrates. The results suggested consuming PUFA is associated with a significant weight difference when replacing SFA (mean change −0.51 kg; 95% confidence interval (CI): −0.81, −0.20), proteins (−0.27 kg; 95% CI: −0.49, −0.04) or carbohydrates (−0.20 kg; 95% CI: −0.37, −0.02). Additionally, isocaloric substitution of 5 EN% of SFA with MUFA was inversely associated with body weight (−0.38, 95% CI: −0.69, −0.07). Increasing SFA at the expense of carbohydrates was found to be directly associated with body weight (0.31 kg; 95% CI: 0.06, 0.56).

#### 3.2.2. Obesity Incidence and Reversion, Substantial Weight Gain and Loss

In a total number of 3700 initially non-obese participants, 657 incident cases of obesity were observed during follow-up. Table 3 shows the results of the logistic GEE investigating the association between nutrient substitution and 2 dichotomous outcomes: Obesity incidence and substantial weight gain (≥10%).

**Figure 1:**
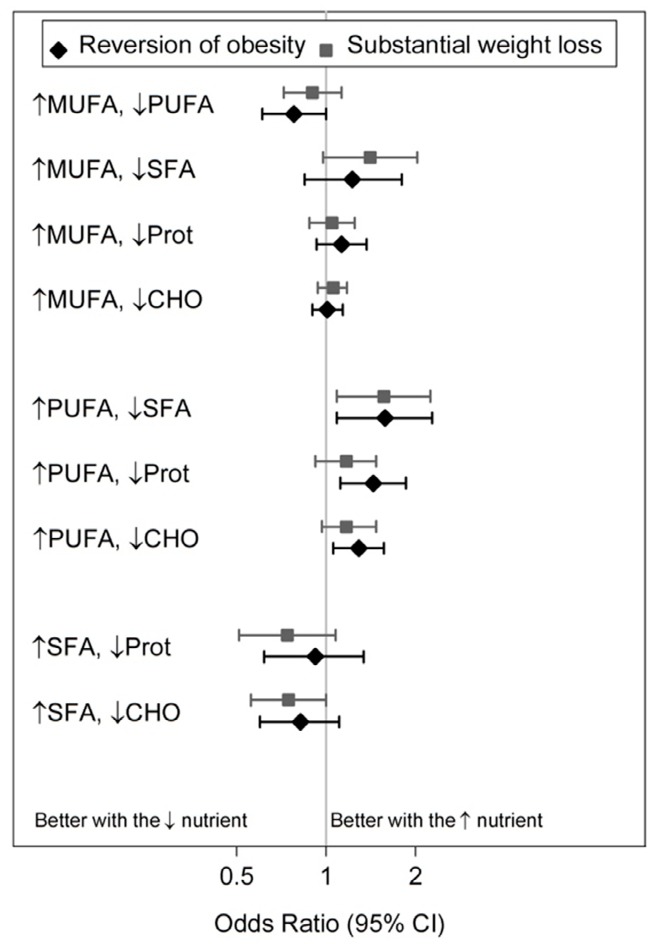
Estimated odds ratios (95% confidence interval) for reversion of obesity (grey squares) and substantial weight loss (≥10%, black diamonds) after isocaloric substitution of 5% energy from MUFA, PUFA, and SFA, proteins (Prot), and carbohydrates (CHO). Adjusted for the same confounders as the analyses in Table 3.

Replacing 5 EN% of proteins with 5 EN% of PUFA decreased the odds of obesity incidence (OR: 0.62, 95% CI: 0.47, 0.83) and ≥10% weight gain (OR: 0.70, 95% CI: 0.50, 0.99). The odds ratio (95% CI) for obesity incidence was 1.36 (1.02, 1.81) when 5 EN% of PUFA was replaced by MUFA. Reversion of obesity occurred 819 times within the 3242 participants who were obese at baseline. Note that in Figure 1 an odds ratio (OR) >1 indicates increased odds of reversion of obesity or weight loss (≥10%), so desirable changes.

Replacing SFA with PUFA significantly increased the odds of the reversion of obesity (OR: 1.57, 95% CI: 1.09, 2.25) and substantial weight loss (OR: 1.58, 95% CI: 1.09, 2.28). Slightly less strong but significant associations were found for the odds of substantial weight loss when PUFA intake was increased at the expense of proteins (OR: 1.44, 95% CI: 1.12, 1.86) or carbohydrates (OR: 1.29, 95% CI: 1.06, 1.57). Increasing MUFA at the expense of PUFA resulted in an OR of 0.78 (0.61, 1.00) for a ≥10% weight loss. As a summary, all significant results in Table 3 and Figure 1 advocate for a higher PUFA intake.

### 3.3. Substitution of Food Items

The estimated effects of non-isocaloric food item substitutions on body weight are shown in Table 4. Significant weight difference estimates were observed when daily replacing a 100 g portion of red meat with white meat (−0.64 kg, 95% CI: −0.94, −0.35), oily fish (−0.75 kg, 95% CI: −1.13, −0.38) or white fish (−0.87 kg, 95% CI −1.17, −0.56).

Replacing a 10 g portion of margarine with vegetable oils other than olive oil resulted in a significant estimate, suggesting a weight gain of 0.26 kg (95% CI: 0.02, 0.50). No significant associations were observed for the replacement of butter and margarine with olive oil or replacing 30 g of mixed nuts with an equivalent portion of walnuts.

Estimates of nutrient substitutions for continuous outcomes were consistent when using mixed-effects models with random intercepts for recruitment center and identity level. Mixed-effects models are a little more conservative, however, thus showing wider confidence intervals (data not shown).

## 4. Discussion

The results of this prospective study suggest that increasing the intake of unsaturated fatty acids (especially PUFA) at the expense of SFA, proteins, and carbohydrates may have beneficial effects on body weight and obesity. Reversing obesity seems to be especially successful when replacing SFA with PUFA, and replacing proteins with PUFA lowers the risk of becoming obese in the first place. Increasing MUFA at the expense of PUFA, on the other hand, shows a significantly increased risk of becoming obese. These findings are in line with previous research [30], although there is still controversy [31]. Most prior research on the association between dietary fat and overweight/obesity merely considered total fat intake or the MeDiet as a whole. Evidence from 53 randomized controlled trials summarized in a systematic review and meta-analysis did not support low-fat diets over other dietary interventions for long-term weight loss, when compared with dietary interventions of similar intensity [15]. A systematic review of 5 RCTs found the MeDiet resulted in greater weight loss than low-fat diet at ≥12 months but produced results similar to other comparator diets, like a low-carbohydrate diet [14]. These results are in line with an earlier meta-analysis [13]. Although the results of the included studies were inconsistent and the meta-analyses reported significant heterogeneity, the evidence ultimately points towards a role of a high-quality, moderately high-fat diet like the MeDiet in the prevention of overweight and obesity. Results of the PREDIMED trial supported that the intervention did not have a differential effect on body weight compared to the control group [32].

One prospective study in a Spanish sample of the EPIC (European Prospective Investigation into Cancer and Nutrition) study further focused on dietary fat subtypes and concluded that the association between consumption of specific types of dietary fat, olive oil, and obesity in Spain was not very important [33]. However, the design of the study was cross-sectional. Ten years later, the results of the EPIC–PANACEA study showed the MeDiet may prevent weight gain and the development of obesity. Unfortunately, this study did not distinguish between types of fat [34].

Previous PREDIMED studies showed a protective effect of MeDiet interventions supplemented by olive oil or nuts and of dairy consumption [35,36]. Moreover, extensive evidence was found that nut consumption does not increase body weight and obesity incidence and may even be inversely associated with these outcomes [17,37,38]. Most nuts are especially high in MUFA, whereas walnuts are rich in PUFA. Adherence to the MeDiet in general has also been shown to be directly associated with fulfilling nutrient recommendations [39,40].

Another important finding of our study, in the context of substitution models, is that reductions in red meat consumption coupled with respective increases in white meat or fish would lead to less weight gain. This is important because it represents a translation of fat substitution models into food item replacement models and it generates a comprehensible and easily understandable message for the population. This is in accordance with the concept of the MeDiet, which fosters the preference for white meat consumption over red meat and includes regular fish consumption. It is also important to focus on specific food groups or items, since different foods rich in a specific fat subtype may have different effects on body weight [41].

The satiating effect of the MeDiet may explain some beneficial effects on body weight, especially in a longer term. Furthermore, evidence shows mono- and polyunsaturated fatty acids are more readily oxidized than saturated fatty acids and have a greater thermogenic effect, which can lead to reduced fat accumulation [42]. The quantity and quality of dietary fat have also been shown to induce differential lipid storage and processing gene expression, influencing adipokines release, but this process and regulation is still only partially understood [43]. Adherence to MeDiet may also be inversely correlated with proinflammatory biomarkers [44], and another potential important mechanism is the influence on adipose tissue metabolism and secretory functions [45].

The major strengths of the present research include its prospective design, large sample size, and repeated measurements of exposure, outcomes, and confounders over a long follow-up period. Due to comprehensive data records, most relevant confounders could be controlled for. Nevertheless, residual confounding is possible. Genetic determinants for instance may influence measures of obesity and were not available in the dataset [46]. Systematic error may be present due to the large proportion of obese individuals, known to have a tendency to underestimate their dietary intakes (in particular total energy intake) and overestimate physical activity; however, this error will probably be nondifferential [47]. Furthermore, well-known measurement errors as a result of methodological issues in the memory-based assessment of dietary factors and physical activity may bias the results. The study of waist circumference as a potential outcome measure was also considered and would shine light on the matter, but the risk of substantial measurement error, especially in overweight and obese participants [48], makes it worthy of special attention and more complex analyses are warranted, so we decided not to include it in this manuscript for the sake of simplicity. Both the FFQ and physical activity questionnaire have been validated, however [22,23,27,28]. Energy-adjusted intraclass correlation coefficients for MUFAs, PUFAs, and SFAs in four 3-day dietary records and the FFQ were 0.61, 0.60, and 0.75, respectively [22]. Analyses of trans fatty acids were considered. Intake of this fat subtype was very low in this study, however (0.47 ± 0.13 EN%); thus, these investigations may be more effective in a different population.

This study cannot fully demonstrate causality of the observed associations. Obesity and body weight may be susceptible to reverse causation in the sense that once their body changes, participants adjust their eating habits [49]. The use of a generalized estimating equation was chosen for all of the analyses. An alternative could have been multilevel mixed-effects models. Both GEE and mixed-effect methods are appropriate for longitudinal data as they account for within-subject correlations occurring as a result of repeated measurements, but GEE models are more flexible. For linear regression models with continuous outcomes, the random effects assumption is equivalent to an exchangeable correlation structure in GEE [50]. The sensitivity analyses confirmed this as estimates were very similar, though the outcomes of the mixed-effects models were more conservative with wider confidence intervals. Models assessing the effect of actual changes in diet on changes in weight may lead to more interpretable results and better conclusions [41].

Although a lot of effort was put into collecting complete data, missing data are unavoidable in longitudinal studies. Throughout the years, this problem has been well documented and a range of solutions have been proposed [29]. In this case, multiple imputation was used only for the GEE model with body weight as a continuous outcome, to verify that the results obtained by complete case analyses with covariate adjustment were indeed unbiased. Multiple imputation is flexible when it comes to assumptions regarding the randomness of the missing data, which makes it suitable for sensitivity analyses in the case of MNAR scenarios. Complete case analysis was used for further analyses as the results were similar and this method is less time-consuming, more transparent, and at least as efficient as multiple imputation [29].

The population of this study consisted of older participants at high risk of cardiovascular disease and (almost all) overweight or obese at baseline, which may affect the generalizability of the findings. The number of subjects was, however, very large, and subjects came from different social levels and geographical areas and biological mechanisms will be similar in other populations. Moreover, mean total fat intake represented 40.5% of the total energy intake in this study population, which is comparable to the intake observed in a pooled analysis of studies across Spain [51]. Mean intakes of fat subtypes were slightly more desirable than intakes measured previously in Spain: MUFA 20.2 in the present study versus 15.9 EN%, PUFA 6.5 versus 5.6 EN% and SFA 10.3 versus 12.0 EN%. 

Lastly, body weight was a secondary outcome in the PREDIMED trial. Neither energy restriction nor increased physical activity were advised for any treatment arm, as the intervention was not designed with the intention of causing weight loss. It should also be noted that one diet does not fit all, and weight control strategies should be based on personal goals. The MeDiet could, however, be a useful method to achieve personal goals, due to its high palatability and satiating effects and, therefore, better adherence [46]. Integrating energy restriction and increased physical activity could further enhance weight loss and health benefits [38]. Currently, data are being collected for the PREDIMED-Plus trial, including an intensive lifestyle intervention program with an energy-restricted MeDiet, increased physical activity, and behavioral treatment.

## 5. Conclusions

In conclusion, this study provides evidence that increasing MUFA and PUFA intakes at the expense of SFA, proteins, and carbohydrate intakes may contribute to losing weight and reverse the state of obesity in an older and overweight/obese Spanish population. It could therefore be recommendable to encourage a high-quality, moderately high-fat eating pattern like the MeDiet, which means increasing the quality of dietary fat instead of restricting total fat intake in the prevention of obesity.

## Figures and Tables

**Table 1:** Participant characteristics according to extreme quintiles of total fat (in EN%) at baseline ^1^.

	Q1	Q5
Participants, n	1391	1387
Age (year)	67 (6)	67 (6)
Women (%)	53.9	62.5
Control group (%)	35.6	32.2
Physical activity (MET-min/week)	249 (259)	216 (226)
Smoking habit: Never smoker (%)	61.0	64.4
Smoking habit: Former smoker (%)	24.4	22.4
Smoking habit: Current smoker (%)	14.6	13.2
Marital status: Married (%)	75.0	76.5
Marital status: Single/widowed (%)	25.0	23.5
Educational level: Lower than high school (%)	80.5	75.3
Educational level: High school (%)	11.9	17.7
Educational level: University (%)	7.6	7.0
Employment status: Employed/housewife	41.6	51.8
Employment status: Unemployed (%)	3.5	3.5
Employment status: Retired (%)	54.9	44.7
Total energy intake (kcal/day)	2262 (573)	2163 (513)
Carbohydrates (EN%)	51.6 (5.8)	35.1 (4.5)
Protein (EN%)	17.4 (3.1)	16.7 (2.8)
Total fat (EN%)	27.7 (3.2)	46.5 (3.7)
MUFAs (EN%)	14.4 (2.4)	26.3 (3.4)
PUFAs (EN%)	5.0 (1.6)	7.9 (2.4)
SFAs (EN%)	8.1 (1.7)	12.1 (2.1)
*trans* fat (EN%)	0.18 (0.12)	0.28 (0.17)
Alcohol (g/day)	11.2 (18.7)	5.1 (9.2)
Dietary fiber (g/day)	28.9 (10.1)	21.7 (6.6)

^1^ Q, quintile; MET, metabolic equivalent task; EN%, energy as a proportion of total energy intake. Data are mean (SD) unless otherwise stated.

**Table 2:** Estimated mean changes (95% confidence interval) ^1^ in body weight (kg) after isocaloric substitutions of 5% energy from MUFA, PUFA, and SFA, proteins, and carbohydrates.

Substitution	↑MUFA	↑PUFA	↑SFA
↓**PUFA**	0.13 (−0.09, 0.34)	-	-
↓**SFA**	−0.38 (−0.69, −0.07) *	−0.51 (−0.81, −0.20) *	-
↓**proteins**	−0.14 (−0.30, 0.02)	−0.27 (−0.49, −0.04) *	0.24 (−0.08, 0.56)
↓**carbohydrates**	−0.07 (−0.17, 0.03)	−0.20 (−0.37, −0.02) *	0.31 (0.06, 0.56) *

^1^ Adjusted for age, sex, baseline weight, recruitment center, intervention group, cumulative average of total energy intake, BMI, leisure-time physical activity (metabolic equivalent task in min/day), smoking status (never, former, current smoker), educational level (primary education, high school, university), working status (employed, unemployed, housewife, retired), and marital status (single, married). * *p* < 0.05. ↑ means increases, ↓ means decreases.

**Table 3:** Estimated odds ratios (95% confidence interval) ^1^ for incidence of obesity and substantial weight gain (≥10%) after isocaloric substitution of 5% energy from MUFA, PUFA, and SFA, proteins, and carbohydrates.

Substitution	Obesity Incidence	Weight Gain (≥10%)
↑MUFA, ↓PUFA	1.36 (1.02, 1.81) *	1.16 (0.82, 1.65)
↑MUFA, ↓SFA	1.04 (0.69, 1.56)	1.11 (0.71, 1.65)
↑MUFA, ↓Proteins	0.85 (0.69, 1.03)	0.82 (0.66, 1.02)
↑MUFA, ↓Carbohydrates	1.08 (0.95, 1.23)	1.02 (0.87, 1.18)
↑PUFA, ↓SFA	0.77 (0.51, 1.15)	0.96 (0.62, 1.47)
↑PUFA, ↓Proteins	0.62 (0.47, 0.83) *	0.70 (0.50, 0.99) *
↑PUFA, ↓Carbohydrates	0.80 (0.63, 1.01)	0.87 (0.67, 1.15)
↑SFA, ↓Proteins	0.81 (0.53, 1.24)	0.73 (0.46, 1.17)
↑SFA, ↓Carbohydrates	1.04 (0.75, 1.24)	0.91 (0.63, 1.31)

^1^ Adjusted for age, sex, baseline weight, recruitment center, intervention group, cumulative average of total energy intake, BMI, leisure-time physical activity (metabolic equivalent task in min/day), smoking status (never, former, current smoker), educational level (primary education, high school, university), working status (employed, unemployed, housewife, retired), and marital status (single, married). * *p* < 0.05.

**Table 4:** Estimated mean changes in body weight (kg) (95% confidence interval) ^1^ after substitution of one daily portion of high-fat food items in the MeDiet for a priori healthier options.

Substitution	Mean Body Weight (kg) Difference (95% Confidence Interval)
↓Red meat, ↑White meat	−0.64 (−0.94, −0.35) *
↓Red meat, ↑Oily fish	−0.75 (−1.13, −0.38) *
↓Red meat, ↑White fish	−0.87 (−1.17, −0.56) *
↓Butter, ↑Olive oil	−0.25 (−0.56, 0.06)
↓Butter, ↑Other vegetable oils	−0.11 (−0.44, 0.22)
↓Margarine, ↑Olive oil	0.04 (−0.18, 0.25)
↓Margarine, ↑Other vegetable oils	0.26 (0.02, 0.50) *
↓Mixed nuts, ↑Walnuts	−0.15 (−0.61, 0.32)

^1^ Substitutions of a portion of 100, 10 and 30 g for meat/fish, butter/margarine/oils, and nuts, respectively. Adjusted for age, sex, baseline weight, recruitment center, intervention group, cumulative average of total energy intake, BMI, leisure-time physical activity (metabolic equivalent task in min/day), smoking status (never, former, current smoker), educational level (primary education, high school, university), working status (employed, unemployed, housewife, retired), and marital status (single, married). * *p* < 0.05. ↑ means increases, ↓ means decreases.
